# The Novel Use of Robot‐Assisted Gait Training in the Treatment of Functional Neurological Disorder: A Case Report

**DOI:** 10.1155/crnm/5007133

**Published:** 2026-08-16

**Authors:** Sam Smith, Lydia Caisley Harland, Bernie Bissett, Tapiwa Matangira, Philip Gaughwin

**Affiliations:** ^1^ Physiotherapy Department, Canberra Health Services, Canberra, Australian Capital Territory, Australia; ^2^ Faculty of Health, University of Canberra, Canberra, Australian Capital Territory, Australia, canberra.edu.au; ^3^ Rehabilitation Medicine, Canberra Health Services, Canberra, Australian Capital Territory, Australia

**Keywords:** case report, functional neurological disorder, Hocoma Lokomat, physiotherapy, robot-assisted gait training

## Abstract

Functional neurological disorder (FND) is a condition defined by neurological symptoms that may be motor, sensory or cognitive in nature and can result in significant functional impairment, including total loss of mobility. There is evidence supporting the use of robotics in other neurological conditions such as stroke and spinal cord injury, but there is currently no published evidence for its use in the treatment of FND. This case using the Hocoma Lokomat robotic exoskeleton device represents the first known case of robot‐assisted gait training in the treatment of FND. The aim of this case study was to determine if robot‐assisted gait training (RAGT) could be a viable treatment option to improve mobility for patients with FND. Over 9 weeks, the 34‐year‐old patient completed 15 RAGT sessions. Outcome measures included the functional ambulation category (FAC), functional independence measure (FIM), 10‐m walk test (10MWT) and Oxford manual muscle strength testing (MMT). At the conclusion of the RAGT programme, the patient had achieved independent walking: FAC improved from 0/5 to 5/5, FIM Locomotion improved from 1/7 to 7/7, FIM Total improved from 56 to 123 and the 10 MWT was completed in 37.45 s. The patient’s muscle strength improved from 0/5 to 5/5 in all lower limb muscle groups (MMT). At 6 months postintervention, the patient reported zero FND relapses or functional impairments. RAGT can be considered a potential intervention option in FND, specifically in cases with gait impairments, and can be associated with dramatic improvements in strength and mobility. This is the first case of a patient with FND successfully completing RAGT in the treatment of gait‐related impairments. Studies exploring feasibility and patient acceptability of this novel therapy in FND are now warranted.

## 1. Introduction

The term ‘functional neurological disorder’ (FND) represents a broad class of common and highly disabling conditions that have a profound impact on physical function, interpersonal relationships, employment and quality of life [1]. Symptoms encompass functional seizures, tremors, weakness, movement and gait disorders, speech impairments and altered sensorium. The condition has a poor long‐term prognosis without rehabilitation and a high rate of recurrence [2]. Effective diagnosis and treatment of FND requires a multidisciplinary, collaborative effort between neurology, psychiatry, rehabilitation medicine and allied health disciplines to establish a definitive diagnosis and endorse a therapeutic approach [3, 4]. Consensus recommendations for physical therapy in FND promote retraining of abnormal movement patterns, education and self‐management strategies [5–7]. The focus of therapy should be on general function and automatic movements such as walking rather than controlled movements targeting specific impairments, for example, strengthening exercises [8]. However, anecdotally, patients with FND can often be challenging to treat, with wide variations in response to therapy.

**FIGURE 1:**
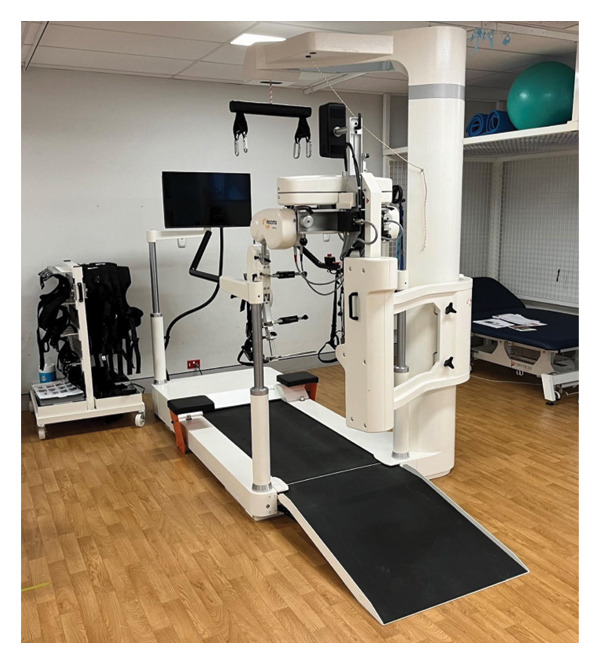
Hocoma Lokomat robotic exoskeleton device in the rehabilitation gym.

Robot‐assisted gait training (RAGT) platforms such as the Hocoma Lokomat (Figure 1) are safe and effective in the neurorehabilitation of stroke [9–11], Parkinson’s disease [12, 13] and spinal cord injuries [14, 15]. Compared with conventional therapy, RAGT delivers a quantitatively higher dosage of repetitive gait training [16]. Most importantly, RAGT platforms also provide rapid, direct and high‐quality biofeedback on gait performance to both therapist and patient [17].

The Hocoma Lokomat is an electromechanical‐assisted exoskeleton device that facilitates gait training on a treadmill by providing adjustable body weight support whilst guiding the patients’ lower limbs through a physiological gait pattern using two actuated orthoses modifiable via the guidance force parameter. The Lokomat device can be programmed by the therapist to provide varying levels of assistance by altering body weight support and guidance force parameters depending on the patient’s needs, therapeutic goals and capabilities [18, 19].

Here, we present a case of a profound and sustained improvement in gait function for a patient with long‐established FND in response to a conservative trial of RAGT in a supervised clinical setting and as part of a multidisciplinary, bio‐psycho‐social inpatient rehabilitation admission. This novel case highlights the critical need for further studies of RAGT and neurorehabilitation interventions for FND.

## 2. Case Presentation

This patient consented to sharing her information in this study. The patient was a 34‐year‐old white female with a past medical history of FND, complex PTSD, borderline personality disorder, dyslipidaemia, obstructive sleep apnoea and depression. She presented to her local hospital with a 4‐day history of right‐sided severe headache, left‐sided weakness, nausea and vomiting. On primary assessment, Glasgow Coma Scale was 13 (E3V4M6) with other vital signs within normal range. She was unable to move any of her four limbs and provided mumbled responses to questions.

She was diagnosed with FND by a neurologist when she lived in another health district. Three previous relapses triggered by psychological stressors were reported. Her symptoms presented as functional hemiplegia, stuttering speech and functional seizures in association with headaches. Symptoms usually resolved within a few days, and she remained independent in function. The symptoms during this presentation were similar but more severe compared to previous episodes of FND relapse. After structural causes of symptoms were ruled out, the clinical diagnosis provided by the treating neurologist was a relapse of FND.

She was admitted for treatment of possible hemiplegic migraine with lignocaine infusion and commenced on regular analgesia and antiemetics. At 24 h into her lignocaine infusion, there was a MET call for generalised seizure activity during which the patient was able to open her eyes and obey commands. During her seizures, her eyes were closed or fluttering, body movement was atypical and there was no postictal state (immediate recovery). These positive signs were in keeping with a functional seizure and managed with supportive care and patient education. The quality of her speech had also changed, with stuttering, difficult or effortful articulation and a soft quality of voice. At this stage, the patient required full nursing care owing to the left‐sided hemiplegia, was requiring a sling hoist for transfers and was incontinent of bladder and bowel. There was no further medical imaging performed.

Prior to this admission, her last FND relapse had been 11 years ago. The psychological trigger for this relapse was a physical assault. Court proceedings and media coverage perpetuated her symptoms during the admission. There was also a history of mental health admissions for medication overdose in the setting of suicidal intent. The patient had recognised psychosocial disability (generalised anxiety disorder) with case management from mental health services. She had limited local social support.

The lignocaine infusion continued for 7 days. Her admission was complicated by recurrent functional seizures of short duration (30 s–5 min), constipation, stuttering speech and residual nausea. She was encouraged to attend ward‐based physiotherapy and provided advice for active self‐management of her headaches and FND. She was referred to clinical psychology and psychiatry for review of current and recent psychological and social stressors.

The patient had participated in ward‐based therapy and was accepted for inpatient rehabilitation in a nearby freestanding rehabilitation teaching hospital (University of Canberra Hospital, UCH). The patient was admitted to UCH 1 month after her initial acute admission.

**FIGURE 2:**
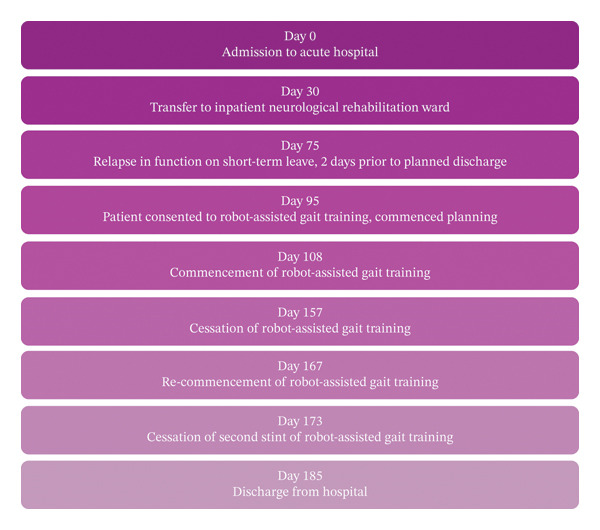
Timeline of patient’s progress during hospital admission.

The patient’s timeline is summarised in Figure 2. FIM score on admission was 41 (Motor 13, Cognition 28) (Table 1). She required full assistance for personal care and required the use of a sling hoist for transfers. Initial neurological assessment at UCH revealed weakness in bilateral lower limbs, demonstrating a 0/5 Oxford manual muscle testing score in all muscle groups. Other symptoms included bilateral lower limb tremors, dysarthria, pain and fatigue.

**TABLE 1:** Functional independence measure (FIM) scores across the inpatient rehabilitation admission.

	Admission	Prerelapse	Pre‐RAGT	Post‐RAGT
	Day 30	Day 72	Day 108	Day 175
Eating	1	7	7	7
Grooming	1	7	5	7
Bathing	1	7	1	7
Dressing upper body	1	7	5	7
Dressing lower body	1	7	1	7
Toileting	1	7	1	7
Bladder management	1	7	1	7
Bowel management	1	7	1	7
Transfer to bed/chair/wheelchair	1	7	1	7
Transfer to toilet	1	7	1	7
Transfer to shower/tub	1	6	1	6
Locomotion	1	7	1	7
Stairs	1	5	1	6
Comprehension	7	7	7	7
Expression	5	6	5	7
Social interaction	5	6	5	6
Problem solving	5	7	5	7
Memory	6	7	7	7
	41	121	56	123

A goal‐setting meeting and comprehensive care plan for inpatient rehabilitation was developed in collaboration with the patient, with the goal to re‐establish independence with mobility and transfers and to retrain personal care for bladder and bowels in the next 6 weeks. This 6‐week plan was formulated in line with the patient’s expected length of stay of 43 days, calculated from Australian National Subacute and Non‐Acute Patient (AN‐SNAP) data. Physiotherapy treatment focused on normalising movement patterns, reducing abnormal self‐directed attention and addressing learned behaviours that contributed to her functional impairments. Occupational therapy was referred for practice of activities of daily living, including meal preparation, dressing and showering. Speech pathology was engaged for management of dysarthria and stutter. A dietician was referred to for disordered eating and weight loss. Counselling and neuropsychology support were contacted for trauma‐informed care support throughout the admission, with the patient receiving weekly psychology sessions.

**FIGURE 3:**
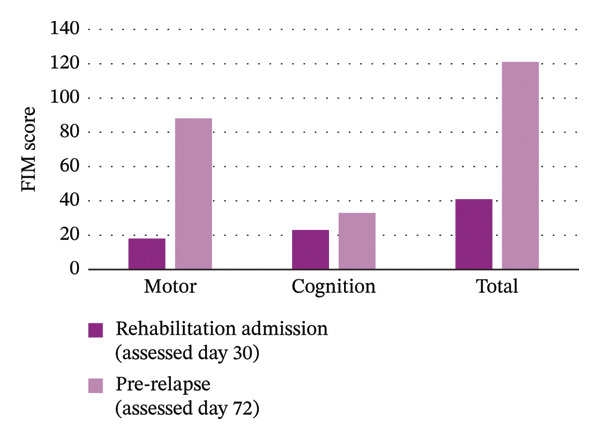
Functional independence measure (FIM) scores during initial inpatient rehabilitation period.

The patient’s response to the agreed multidisciplinary programme was initially positive. She was independent with her mobility and personal care within the agreed‐upon time frame, with a FIM score of 121 (Table 1, Figure 3) and began preparing for discharge from hospital with periods of short‐term leave from the hospital.

A supervised occupational therapy home visit assessed the patient’s suitability for discharge. She managed most personal care tasks independently and could safely access key areas of the home. However, the only exit from her home was a steep, noncompliant ramp, which she could not negotiate independently, requiring standby assistance. In the 24 h before an overnight leave trial, she reported worsening urinary symptoms but did not seek treatment or escalation. She went home Friday night with family support for most of Saturday. That night, a domestic dispute next door left her hypervigilant and sleep‐deprived. The resulting fatigue impaired her mobility the following day. At approximately 9 p.m., she contacted the hospital reporting a severe functional relapse at home. Although advised to return if unsafe, she required assistance from friends and family to exit the property, causing a two‐hour delay. Subsequent review identified contributing factors including social isolation, possible untreated urinary infection, inability to exit the home independently, neighbour conflict, fatigue and poor sleep.

On reassessment, she showed clear functional bilateral lower limb weakness with positive signs such as tremor entrainment. While she could activate quadriceps muscles supine, she was unable to produce movement. In sitting, cocontraction and shaking were observed during sit‐to‐stand, with inability to achieve full extension despite maintaining a semisquat. Functionally, she required a sling hoist for transfers and a wheelchair for all mobility. Her FIM score was 56 (Motor 27, Cognition 29) (Table 1). The following day, lower limb strength was 0/5 bilaterally on MMT, though upper limb function remained intact, allowing independent feeding and upper body dressing.

Following her relapse, she recommenced the original treatment plan which had previously resulted in independent mobility with increased counselling support. After an initial period of 20 days without improvements in function, alternative neurorehabilitation strategies were explored. Noting her prior positive response in previous FND relapses, hydrotherapy was considered but discounted owing to her persistent incontinence of bladder and bowel. Conventional body weight support treadmill training was also determined to be unsuitable due to the rapid onset of functional seizures on the sit‐to‐stand movement and manual handling risks to the therapists. Therefore, the feasibility of completing body weight supported gait retraining using the Hocoma Lokomat robotic exoskeleton therapy device was investigated.

**FIGURE 4:**
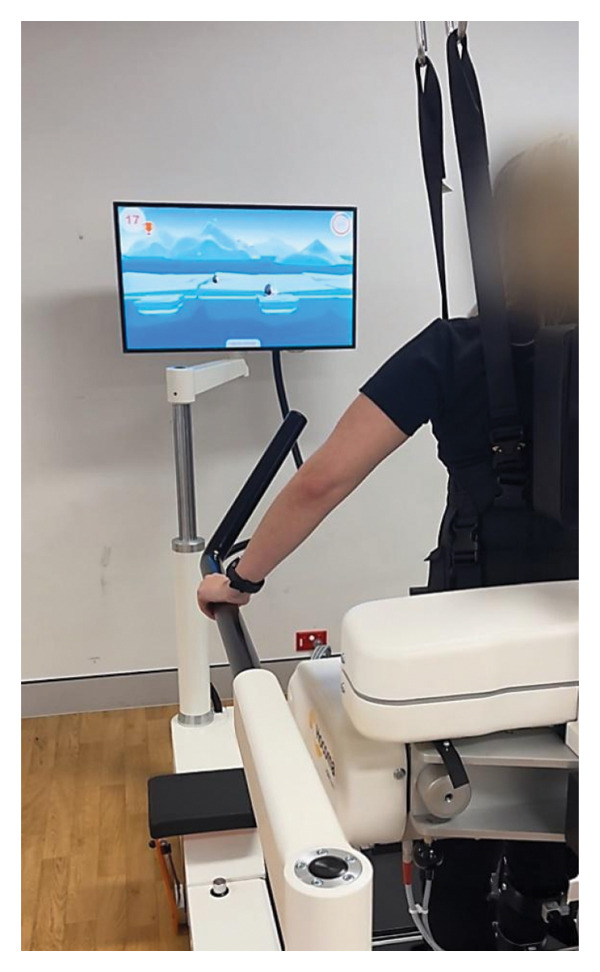
Patient view of screen while using the Hocoma Lokomat robotic exoskeleton device.

A useful diagnostic feature of FNDs is distractibility, meaning the decrease in severity of symptoms in response to redirection of attention away from a task or movement [4, 5, 20, 21]. The Hocoma Lokomat robotic exoskeleton device facilitates mobility by retraining a normal gait pattern, while attention is directed towards external cues, such as the patient’s avatar, games, targets and music (Figure 4). Rather than directly focus concentration on an individual limb or movement, the patient can see their performance represented in the performance of their avatar in the games through various outcomes including accruing more points, increasing speed or changing direction. We posited that the Lokomat’s advantageous form of distraction, linked to indirect engagement with an RAGT‐based physical neurorehabilitation programme, could potentially help this patient regain the ability to reproduce consistent high‐quality voluntary movement. In this sense, RAGT aligned with FND physiotherapy consensus recommendations [7], wherein effective treatment should focus on completing functional task practice while diverting attention away from abnormal movement.

**FIGURE 5:**
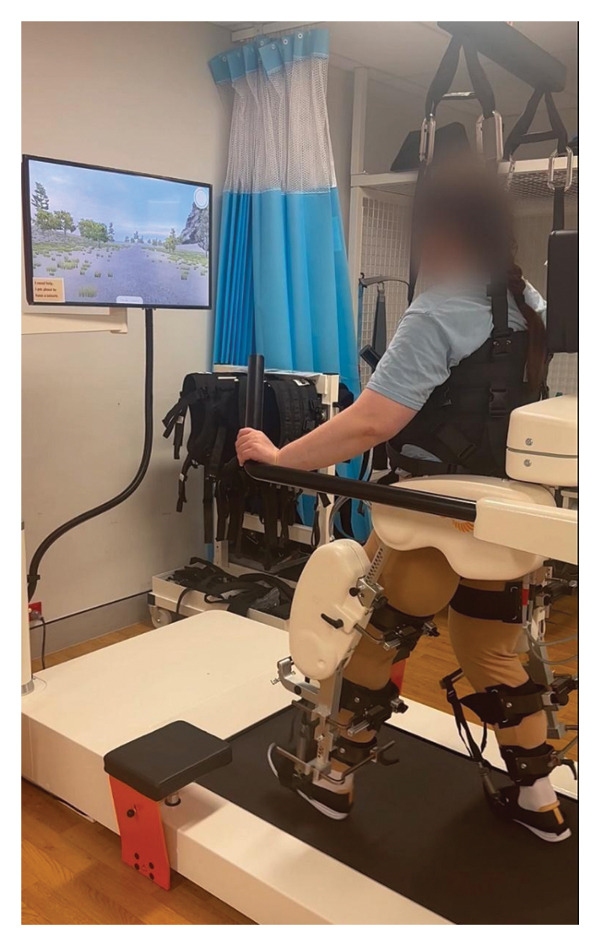
Patient using the Hocoma Lokomat robotic exoskeleton device.

Prior to practical application, considerable time and effort was invested in building acceptability and familiarity with the intervention. Prior to commencing her first RAGT session, the patient observed other patients completing therapy on the Lokomat over a 2‐week period to gain a better understanding of how it operated. The first two sessions were completed without the use of the Lokomat orthosis and games to enable the patient to build comfort with the harness and treadmill. In these first two sessions, two therapists manually guided one leg each for the patient on the treadmill, which is similar to conventional body weight‐supported treadmill training, while a third therapist was responsible for the controls. The first session lasted 2 min and 30 s, and the patient walked 24 m. The second session lasted 20 min, and the patient walked 186 m. For the third session, the patient felt confident in trialling the orthosis. This trial proved successful, and all future sessions were completed using the orthosis (Figure 5). At the conclusion of the first Lokomat session, the patient was able to independently lift her feet onto the foot plates of her wheelchair when being transferred off the Lokomat. This was the first active lower limb movement the patient had demonstrated since her relapse, indicating the potential for RAGT to improve this patient’s function.

The patient completed 15 RAGT sessions during her admission. The first 11 sessions occurred across a period of 7 weeks. After 11 sessions, the patient ceased RAGT to focus on overground walking as the patient had achieved independent walking indoors. Ten days later, RAGT was recommenced as the patient had become distressed with a lack of functional progress since ceasing RAGT. The patient attributed her previous progress to the RAGT and believed it could help her achieve greater walking performance to achieve her goal of independent walking for all community ambulation. The patient completed a further four RAGT sessions over a 6‐day period, at which point she agreed to cease RAGT to concentrate on overground walking and other functional tasks to prepare for discharge from hospital. Across all 15 sessions, the patient completed 5 h 11 min and 53 s of RAGT and covered 7583 m.

The patient was given the opportunity to determine her therapy plan for each session, including dosage, duration and games used. The patient typically completed between three and five games per session on the Lokomat. The patient was given the choice of which games to play, length of each game and after each game was asked if she wanted to continue with another game or end the session.

The patient had suffered multiple functional seizures whilst participating in conventional FND therapy. These were brought on when the patient attempted to actively move her lower limbs or stand from a chair. Seizures are considered a risk factor by Hocoma, the manufacturer of the Lokomat, rather than a contraindication. The risks were weighed against the potential benefits of completing RAGT. An individualised seizure management plan was created by the medical team to follow during physiotherapy sessions. Appropriate risk mitigation strategies were applied to reduce the risk, such as not using the orthosis in the initial sessions, while an additional staff member was on hand for each session to assist with any potential emergency situations. The patient was educated on the risks and benefits of RAGT and consented to participation. The Lokomat has an emergency stop function that allows the session to be stopped immediately. All staff members were trained in emergency procedures for the Lokomat, including removing the patient from the Lokomat in the event of an emergency, and were educated on the patient’s early warning signs and symptoms for a functional seizure. The patient was given key words to notify the clinicians if she felt the early warning signs of a functional seizure. Sessions were also completed outside of regular gym hours so that other patients were not present at the time. Across the 15 sessions, the patient had no instances of functional seizures during RAGT sessions, and the functional seizures also stopped during functional task practice following the implementation of the RAGT. The patient had one instance of discomfort from the harness when attempting to complete RAGT. Despite multiple attempts to reposition the harness, the patient still reported discomfort from the harness, so the session was ceased without completing any games.

**FIGURE 6:**
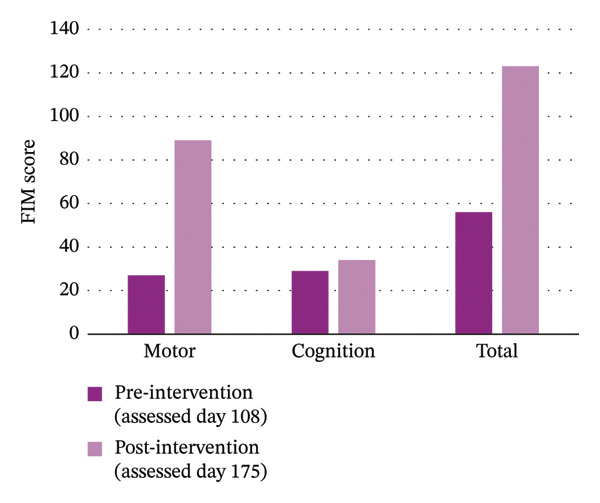
Functional independence measure (FIM) scores pre‐ and post‐RAGT intervention.

The functional ambulation category (FAC) and functional independence measure (FIM) Locomotion outcome measures were used to gauge the patient’s progress. The patient’s FAC improved from 0 to 5 following RAGT, and their FIM Locomotion score improved from 1 to 7. The patient’s scores reflected their progression from being unable to walk to being able to walk independently indoors and outdoors without a gait aid. Their FIM total score improved from 56 prior to RAGT to 123 at discharge (Figure 6). In addition to mobility improvements, the patient achieved independence in her personal cares such as toileting, dressing and bathing (Table 1) and her speech returned to normal. On completion of RAGT, the patient completed the 10MWT independently in 37.45 s, whilst Oxford manual muscle testing in all lower limb muscle groups improved from 0/5 to 5/5 on discharge.

At 6‐month telehealth follow‐up, the patient reported zero FND relapses and that she had returned to her baseline level of function. She reported walking independently without any gait aid in the community and was taking her new dog on walks around her neighbourhood. The following quotes from the patient express her progress in her own words:

“The Lokomat was life‐changing for my recovery ….it helped things ‘click’ in my brain”

“I now have a dog and am able to take my dog on walks.”

## 3. Discussion

This case demonstrates the potential of RAGT as a platform for rehabilitation in FND. We provide a structured approach to the introduction of RAGT to FND patients to promote acceptance, tolerance and enthusiasm for the intervention. The gradual introduction of RAGT appeared to be an important factor in the successful implementation of the therapy. Facilitating opportunities for the patient to observe other patients completing RAGT prior to her first session enabled the patient to develop an understanding of how the device worked. Completing initial sessions without the orthosis component of the Lokomat enabled the patient to build a tolerance to the gait harness and treadmill as well as being in a standing position. The absence of the orthosis also meant the patient could be assured that if she needed to be taken off the device quickly in the instance of a functional seizure, this could occur.

Allowing the patient the autonomy to decide when she felt ready to start using the orthosis was another important factor in the success of the RAGT. The patient was also included in determining dosage and games used during the sessions. Giving the patient autonomy in decision‐making regarding their therapy has been shown to lead to improved outcomes in other patient groups [22] but may be even more important in FND where psychological influences can be profound.

Another potential factor enabling the successful implementation of therapy was the consistency in therapists working with the patient during RAGT. Establishing a core therapy team allowed for a strong rapport and trust from the patient. Having a trusting relationship between the patient and healthcare professionals is key to seeing progress in the patient’s rehabilitation [23]. The patient had belief in the therapist’s judgement that RAGT could lead to improvements in her function. The patient also had confidence that the therapists had a good understanding of her symptoms and warning signs for functional seizures, so would be able to respond quickly in the event of a functional seizure occurring during therapy.

The application of robotics therapy in FND in this case required a pivot in clinical reasoning that would usually be applied to other patient groups. In stroke rehabilitation, best practice gait retraining involves reducing body weight support and external levels of assistance as much as possible, with one study recommending a limit of 30% body weight support not be exceeded due to a reduction in muscle activity [24]. However, in RAGT, the body weight support and guidance force parameters have little effect on muscle activity in stroke patients, with only increased treadmill speeds resulting in increased muscle activity [19]. Our case demonstrated that maintaining a high level of body weight support (50%–80%) and guidance force (50%–100%) from the Lokomat still translated into functional improvement in overground walking. This could be attributed to the patient being able to normalise walking again with the assistance of the increased support of the Lokomat, which provides tailored software adjustments to achieve an optimal gait pattern. As soon as she was able to re‐establish normal walking patterns on the Lokomat, she was able to translate this directly into overground walking without body weight support. This rapid progress aligns with previous advice in FND therapeutic management to emphasise the quality of movement rather than quantity [8].

Since this patient’s admission, the recently published National Institute for Health and Care Excellence guideline NG252 [25] has endorsed the use of robotic exoskeletons for individuals with neurological conditions affecting mobility. Additionally, it recommends that individuals with FND be offered goal‐oriented movement activities with redirection away from symptoms. The successful implementation of RAGT using the Lokomat demonstrates this is possible, aligning with the recommendations from the new guidelines and providing justification for further research in this area.

While there is a lack of validated objective outcome measures that are specific to FND [26], the 10MWT is a standardised walking outcome measure commonly used in other neurological conditions [27, 28]. The patient demonstrated independent mobility to complete an unaided 10MWT at the conclusion of RAGT, although the final speed was still below recommended safe community ambulation speeds [29]. Nonetheless, at 6‐month telehealth follow‐up, the patient reported she had returned to baseline walking speed and was functioning well in the community. Given that attention can exacerbate symptoms, clinical assessments such as the 10MWT may not accurately reflect the patient’s true performance [30] but can demonstrate the initial success of RAGT in this case. The patient was observed to be able to mobilise with greater speed when not being directly assessed, and the patient also used pacing strategies to minimise overexertion. The FIM is a reliable instrument for assessing daily living skills in people with disabilities [31]. The patient saw substantial improvements in FIM scoring across a number of domains, not just limited to locomotion scores, as the patient’s improvements in mobility translated to improvements across these other domains (Table 1).

The use of high‐cost technologies such as RAGT in FND rehabilitation raises important ethical considerations related to equity and allocation of resources. Individuals with FND have historically experienced stigma and marginalisation within healthcare systems, often resulting in reduced access to specialist rehabilitation services [32]. While the use of high‐cost technologies should be balanced against the broader demands of the healthcare service, eligibility for these treatment methods should be based on clinical reasoning and avoid inequities caused by the stigma surrounding conditions such as FND. This case demonstrates the potential for the use of RAGT in this population and provides justification for individuals with FND to be given the opportunity to access this technology where it is available.

The main limitation of this study is that our findings relate to the case in question and may not be applicable to all patients with FND due to the wide variety of presentations observed in this condition. Interestingly, there is considerable evidence that external physical co‐stimulation that builds on mind‐body connection, such as eye movement desensitisation and reprocessing, is an effective treatment for PTSD. More generally, physical activity mitigates stress and anxiety, improves sleep and enhances positive cognitive processes. Therefore, this patient’s underlying mental health comorbidity may have been uniquely responsive to this intervention. This case study does, however, suggest that RAGT may be viable for physiotherapy treatment of FND, particularly for patients with impairments related to transfers and gait. Careful patient selection and individualised therapy are required in all treatment plans for FND [5], and RAGT could be given consideration where this is available.

Second, a set treatment protocol was not used but rather each session’s games, dosage and duration were guided by the patient. Future research could assess the feasibility and efficacy of specific treatment protocols and also explore the acceptability of robotic technology to patients with FND. Finally, access to robotic technology may be a limiting factor in other facilities where these devices are not yet standard in inpatient rehabilitation.

This case demonstrates that RAGT can be a viable intervention for a patient with FND admitted for inpatient rehabilitation, resulting in clinically meaningful improvements in mobility over a period of 9 weeks. This case highlights the potential for RAGT to enhance rehabilitation outcomes in severe FND where traditional therapy may have limited success. Future studies should explore the efficacy, acceptability, safety and long‐term outcomes of robotic therapy in FND rehabilitation to establish evidence‐based guidelines for clinical practice.

## Author Contributions

Sam Smith: conceptualisation, methodology, investigation, data curation, formal analysis, visualisation, resources, writing–original draft, writing–reviewing and editing and project administration. Lydia Caisley Harland: conceptualisation, methodology, investigation, formal analysis, visualisation, writing–original draft and writing–reviewing and editing. Bernie Bissett: conceptualisation, formal analysis, visualisation, writing–reviewing and editing and supervision. Tapiwa Matangira: conceptualisation, methodology, formal analysis, writing–original draft and writing–reviewing and editing. Philip Gaughwin: conceptualisation, methodology, formal analysis, resources, writing–original draft, writing–reviewing and editing and supervision.

## Funding

There was no specific funding for this research, and it was conducted as part of clinical work at the University of Canberra Hospital.

Open access publishing facilitated by University of Canberra, as part of the Wiley University of Canberra agreement via the Council of Australasian University Librarians.

## Disclosure

This study was presented as an oral presentation at a local scientific meeting (Canberra Health Annual Research Meeting) in June 2025^1^ and at the World Congress of Neurology (South Korea) in October 2025, the abstract of which was published in the Journal of Neurological Sciences^2^.

## Consent

Written informed consent was obtained from the patient for publication of this case report including accompanying images.

## Conflicts of Interest

The authors declare no conflicts of interest.

## Endnotes

^1^ https://www.act.gov.au/__data/assets/pdf_file/0010/2842723/CHARM-2025-oral-presentation-abstract-summaries.pdf].

^2^The novel use of robot‐assisted gait training in the treatment of functional neurological disorder: A case report—Journal of the Neurological Sciences.

## Data Availability

Data sharing is available on reasonable request from the corresponding author, in line with patient permissions.
